# Methylone and MDMA Pharmacokinetics Following Controlled Administration in Humans

**DOI:** 10.3390/ijms232314636

**Published:** 2022-11-23

**Authors:** Lourdes Poyatos, Alfredo Fabrizio Lo Faro, Diletta Berardinelli, Giorgia Sprega, Sara Malaca, Simona Pichini, Marilyn A. Huestis, Esther Papaseit, Clara Pérez-Mañá, Francesco Paolo Busardò, Magí Farré

**Affiliations:** 1 Servei de Farmacologia Clínica, Hospital Universitari Germans Trias i Pujol (HUGTiP, IGTP), Universitat Autònoma de Barcelona, 08916 Badalona, Spain; 2 Department of Excellence-Biomedical Sciences and Public Health, Università Politecnica delle Marche, 60121 Ancona, Italy; 3 National Centre on Addiction and Doping, Istituto Superiore di Sanità, 00161 Rome, Italy; 4 Institute of Emerging Health Professions, Thomas Jefferson University, Philadelphia, PA 19107, USA

**Keywords:** methylone, MDMA, pharmacokinetics, humans, LC-MS/MS

## Abstract

The aim of this study is to define, for the first time, human methylone and HMMC plasma pharmacokinetics following controlled administration of 50–200 mg methylone to 12 male volunteers. A new LC-MS/MS method was validated to quantify methylone, MDMA, and their metabolites in plasma. The study was a randomized, cross-over, double-blinded and placebo-controlled study, with a total of 468 plasma samples collected. First, 10 µL of MDMA-d_5_, MDA-d_5_ and methylone-d_3_ internal standards were added to 100 µL of plasma. Two mL of chloroform and ethyl acetate 9:1 (*v*/*v*) were then added, mixed well and centrifuged. The supernatant was fortified with 0.1 mL acidified methanol and evaporated under nitrogen. Samples were reconstituted with a mobile phase and injected into the LC-MS/MS instrument. The method was fully validated according to OSAC guidelines (USA). Methylone plasma concentrations increased in a dose-proportional manner, as demonstrated by the increasing maximum concentration (Cmax) and area under the curve of concentrations (AUC). Methylone Cmax values were reported as 153, 304, 355 and 604 ng/mL, AUC0–24 values were reported as 1042.8, 2441.2, 3524.4 and 5067.9 h·ng/mL and T_1/2_ values as 5.8, 6.4, 6.9 and 6.4 h following the 50, 100, 150 and 200 mg doses, respectively. Methylone exhibited rapid kinetics with a Tmax of 1.5 h for the 50 mg dose and 2 h approximately after all the other doses. HMMC exhibited faster kinetics compared to methylone, with a Cmax value that was 10–14-fold lower and an AUC0–24 value that was 21–29-fold lower. Methylone pharmacokinetics was linear across 50–200 mg oral doses in humans, unlike the previously described non-linear oral MDMA pharmacokinetics. An LC-MS/MS method for the quantification of methylone, MDMA and their metabolites in human plasma was achieved. Methylone exhibited linear pharmacokinetics in humans with oral doses of 50–200 mg.

## 1. Introduction

Synthetic cathinones are a class of novel psychoactive substances (NPS) that are structurally similar to cathinone, a naturally occurring psychoactive molecule from Catha edulis plants [1]. Among the most common cathinones are the 3,4′-methylenedioxy-N-alkylates, which are synthesized by adding a methylenedioxy group to the aromatic ring [2]. This produces substances similar to MDMA or ecstasy in chemical structure and psychotropic effects and resulted in their emergency placement into schedule I of the Controlled Substances Act in 2011 by the US Drug Enforcement Administration (DEA) [3].

3,4-methylenedioxy-methcathinone, more commonly known as methylone or MDMC, is a designer drug of the phenethylamine class that was first identified in 2009. The only structural difference between methylone and MDMA is the replacement of two hydrogen atoms with an oxygen atom in the β position of the phenylethylamine nucleus, forming a ketone group [4]. There are substantial pharmacological similarities between methylone and MDMA, including inhibition of neuronal reuptake of the monoamines dopamine and serotonin and increasing monoamine concentrations in the synaptic cleft [5,6]. In addition, MDMA, methylone and their metabolites displayed interaction with human monoamine transporters. Their capacity to inhibit their uptake may contribute to the effect of the parent drugs on the monoaminergic system, and consequently, to pharmacological effects in humans [7].

There are very few studies of the pharmacological effects of methylone in humans that show similar actions compared to MDMA [8].

For this reason, psychoactive effects, adverse health risks, and signs of intoxication following methylone intake are likely to be similar to those of MDMA [9].

Methylone is mainly metabolized in the liver, with N-demethylation producing 3,4-methylenedioxycathinone (MDC). Methylone is also O-demethylated to 3,4-dihydroxy-N-methylcathinone (HHMC) and the latter is subsequently O-methylated to 4-hydroxy-3-methoxy-N-methylcathinone (HMMC), a primary metabolite [10,11].

There is some evidence in animals for non-linear methylone pharmacokinetics similar to MDMA [10,12,13], due to the multiple structural and metabolic similarities of the two compounds. However, whether or not methylone pharmacokinetics are non-linear was not fully investigated.

The aim of this research is to define methylone’s and HMMC’s human pharmacokinetics and evaluate whether or not non-linear pharmacokinetics occur following controlled administration. To the best of our knowledge, this is the first study on the pharmacokinetics of methylone in humans and the first analytical assay for the simultaneous determination of methylone, MDMA and their related metabolites (HMMC, HMMA, HMA, and MDA). A new high-performance liquid chromatography tandem mass spectrometry (LC-MS/MS) method was developed and validated to quantify methylone, MDMA and their metabolites in human plasma after controlled methylone and MDMA administration.

## 2. Results

### 2.1. Method Development and Validation

**Figure 1:**
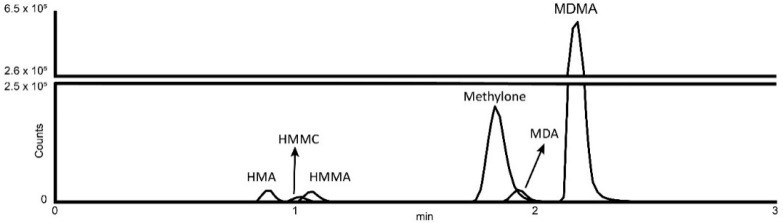
Extracted ion chromatogram of methylone, MDMA and their metabolites. Abbreviations: HMMC, 4-hydroxy-3methoxymethcathinone; MDMA, 3,4-methylendioxymethamphetamine; MDA, 3,4-methylendioxyamphetamine; HMA, 4-hydroxy-3-methoxyamphetamine; HMMA, 4-hydroxy-3methoxymethamphetamine.

The best fit calibration model was a linear least-squares regression model with 1/×2 weighting, as confirmed by Mandel test coefficients. All coefficient of determination results were greater than or equal to 0.99. Linearity, calibration results, LOD and LOQ are shown in Table 1. LOQ was set as the lowest non-zero calibrator for each analyte (Figure 1). There was no carryover observed with any of the analytes.

Accuracy and imprecision were calculated at the following three QC concentrations (n = 5): 7.5, 150 and 350 ng/mL for MDMA and methylone; 1.5, 20 and 80 ng/mL for HMMC, MDA, HMMA and HMA. Bias was <20% of the target. The ANOVA approach defined by OSAC guidelines determined the overall within- and between-run imprecision [14]. All CV values were less than 20%, as shown in Table 1.

There were no interfering peaks in any of the biological matrix pools analyzed. Dilution integrity was evaluated by extracting plasma samples with concentrations two times the ULOQ and diluting the samples 2-, 5-, 10- and 20-fold in blank plasma. Concentrations of replicates (n = 5) for the diluted samples were within ±20% of the target for all compounds.

All analytes were stable at room temperature for 24 h, when refrigerated (4 °C) for 24 h, after three freeze/thaw cycles, 24 h post-extraction in the autosampler (+10 °C) and when stored at −20 °C up to 4 months after QC sample preparation (concentration differences less than 20% with respect to time zero response). Matrix effects were evaluated at low (1.5 and 7.5 ng/mL), mid (20 and 150 ng/mL) and high (80 and 350 ng/mL) concentrations. The post-extraction addition method determined ionization suppression (negative value) or ionization enhancement (positive value) within ±20% for all analytes.

### 2.2. Pharmacokinetics of Methylone, MDMA and Their Metabolites

**Figure 2:**
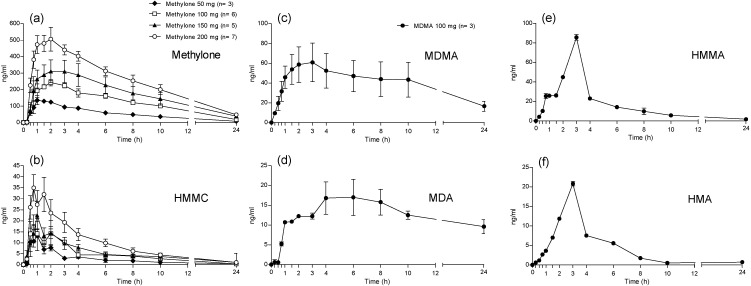
Time–concentration profiles for plasma (**a**) methylone; (**b**) HMMC; (**c**) MDMA; (**d**) MDA; (**e**) HMMA and (**f**) HMA. Error bars correspond to standard error.

Figure 2 shows the time–concentration profiles for plasma methylone and HMMC after oral administration of 50, 100, 150 and 200 mg of methylone and MDMA, MDA, HMMA and HMA after oral administration of 100 mg of MDMA. The pharmacokinetic parameters derived from the data depicted in Figure 2 are presented in Table 2.

**Figure 3:**
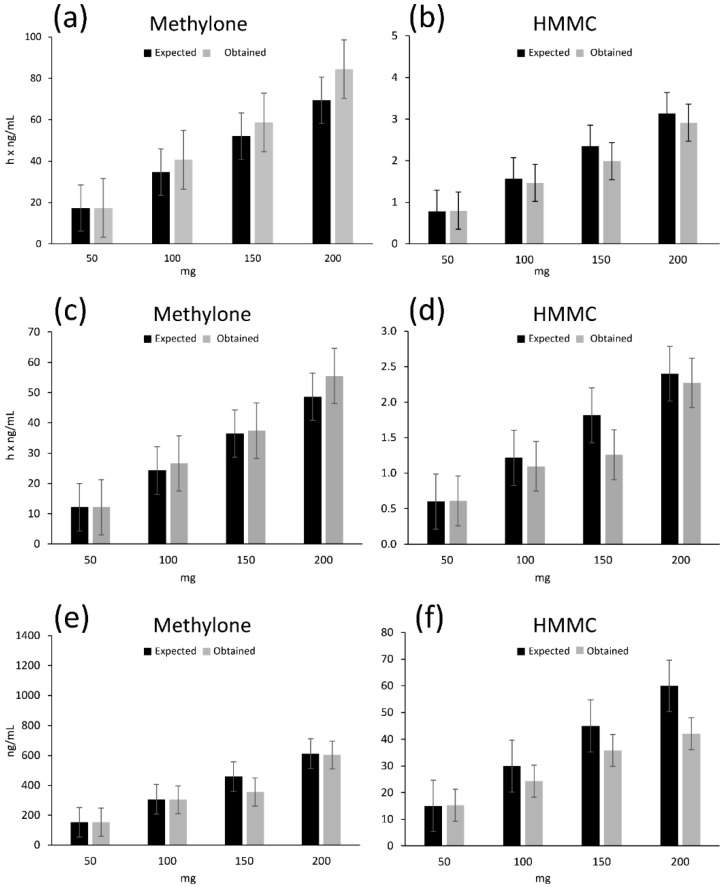
Comparison of expected vs. observed (**a**,**b**) area-under-the-curve 0–24 values (AUC0–24); (**c**,**d**) area-under-the-curve 0–10 values (AUC0–10); (**e**,**f**) maximum concentration (Cmax) values of methylone and HMMC, respectively. HMMC, 4-hydroxy-3methoxymethcathinone.

As shown in Figure 3, the methylone plasma concentrations increased in a dose-proportional manner, as demonstrated by the increasing Cmax and AUC values. Methylone Cmax values were reported as 153.0, 304.0, 355.0 and 604.0 ng/mL following the 50, 100, 150 and 200 mg doses, respectively, while the corresponding AUC0–10 values were 729.2, 1596.2, 2245.6 and 3329.1 h·ng/mL and the AUC0–24 values were 1042.8, 2241.2, 3524.4 and 5067.9 h·ng/mL. Methylone exhibited rapid kinetics with a Tmax of 1.5 h for the 50 mg dose and 2 h approximately after all other doses. Twenty-four hours after drug administration (Tlast), the methylone concentrations were 8.0, 18.8, 38.6 and 47.8 ng/mL following the 50, 100, 150 and 200 mg methylone doses, respectively, yielding T1/2 values of 5.8, 6.4, 6.9 and 6.4 h, respectively.

For methylone’s metabolite HMMC, the Cmax values were 10–14-fold lower, the AUC0–10 values were 19–24-fold lower and the AUC0–24 values were 21–29-fold lower than those of methylone. HMMC Cmax occurred between 0.9 and 1.5 h. Clast concentrations were 0.5, 0.4, 1.3 and 0.8 ng/mL at 24 h, respectively.

Cmax for the 100 mg oral MDMA dose was 66.1 ng/mL at 2.0 h (Tmax), AUC0–10 was 468.2 h·ng/mL and AUC0–24 was 888.6 h·ng/mL. For MDMA metabolites, MDA Cmax was 21.9 ng/mL after 4.0 h, AUC0–10 was 135.9 h·ng/mL and AUC0–24 was 290.8 h·ng/mL. HMMA Cmax was 85.6 ng/mL at a Tmax of 3 h and AUC0–24 was 294.1 h·ng/mL with a T1/2 value of 5.4 h and at 24 h, this reduced to a concentration (Clast) of 1.8 ng/mL. Finally, HMA Cmax was 20.8 ng/mL with a Tmax of 3 h and AUC0–24 was 71.0 h·ng/mL, with a T1/2 of 5.3 h and Clast of 0.7 ng/mL after 24 h. HMMC concentrations were approximately 20 times lower than methylone’s drug concentration, for all administered doses (50, 100, 150 and 200 mg), while HMA, HMMA and MDA concentrations were 8-, 2- and 8-times lower than MDMA’s concentrations, respectively. The relative concentrations of MDMA and its metabolites were MDMA > HMMA > MDA > HMA.

The dose response based on Cmax for the 50, 100, 150 and 200 mg oral doses of methylone resulted in dose-proportional values of 3.1, 3, 2.4 and 3, respectively, and based on AUC0–24, a similar proportionality was observed for the values of 20.8, 24.4, 23.5 and 25.3. In contrast, de la Torre et al. showed non-dose-proportional AUC values for oral 50, 75, 100, 125 and 150 mg MDMA doses of 9.15, 17.8, 18.5, 21 and 34.5, respectively, confirming the non-linear pharmacokinetics of MDMA [12].

## 3. Discussion

The analytical method was fully validated over five consecutive days for the simultaneous quantification of methylone, MDMA and their metabolites and later applied to authentic samples. Plasma samples were collected from twelve male participants after administration of controlled doses of methylone and/or MDMA. Overall, this rapid and simple HPLC-MS/MS method enabled robust and sensitive quantification of methylone, MDMA and their metabolites, with good precision, accuracy, and efficiency. All analytes were stable at room temperature, 4 °C and −20 °C for 24 h, and also after three freeze/thaw cycles, demonstrating the applicability of the validated method for routine analysis.

As expected, methylone concentrations were dose-proportional, at least at the doses administered from 50 to 200 mg. Methylone concentrations were higher than MDMA concentrations at the same 100 mg dose; however, not all participants received all doses (n = 3). Regarding metabolites, at a 200 mg methylone dose, HMMC concentrations were approximately 20-times lower that the parent drug concentrations, HMMA concentrations were 16-fold lower than the parent drug, while MDA concentrations were 3-times lower than MDMA after the 100 mg dose.

Previous studies reported that methylone is extensively metabolized in a manner similar to its structural analog MDMA, as illustrated by the formation of *O*-demethylated metabolites (such as HMMC). The pharmacokinetics of MDMA were studied in both humans and animal models, and it was widely demonstrated that MDMA concentrations exhibit non-linear behavior, due to the inhibition of its own metabolism [14,15,16]. By contrast, Elmore et al. found non-linear pharmacokinetics in rats after subcutaneous administration of methylone at doses of 3, 6 and 12 mg/kg [9]. Unlike its structural analog, linear pharmacokinetics were observed for methylone and its more abundant metabolite (HMMC) in humans, as shown here. It was also verified that plasma concentrations of methylone and its metabolite displayed more rapid kinetics when compared with MDMA [9,10]. Methylone is a common drug of abuse, but to date, only a few studies have addressed its pharmacokinetics and metabolism in animal models [10,17].

For the first time, we investigated the pharmacokinetics of methylone and HMMC in humans and documented linear pharmacokinetics based on normalized C_max_ and AUC values. However, the observed variance is most likely related to individual variability and different participants who received the 50, 100, 150, 200 mg doses of methylone. Comparison of the normalized AUC_0–10_ and AUC_0–24_ values obtained in this study (14.5, 15.9, 14.9, 16.6 and 20.8, 24.4, 23.5, 25.3, respectively) with those obtained by de la Torre et al. for MDMA (9.15, 17.8, 18.5, 21 and 34.5) further supports the thesis of methylone linear pharmacokinetics. The elimination half-life of methylone was 6 h, considerably higher than other cathinones, such as mephedrone (2,3 h) or cathinone (4 h), but considerably less than other phenethylamines, such as MDMA (around 8 h) or amphetamine–methamphetamine (12 h) [18].

A limitation of the present study is that only one methylone metabolite, HMMC, was measured. Unfortunately, nor MDC or HHMC could be determined due to the unavailability of pure chemical standards at the time of the study.

There are few published reports on methylone intoxication [18,19], but given the pharmacological similarity between methylone and MDMA reported in an observational study, there most likely are toxicological effects [20,21]. The toxicological consequences of methylone non-linear pharmacokinetics in animals are poorly understood. In humans, the enzyme CYP2D6 is inhibited by MDMA, and in animals, methylone results in the same inhibition. Our results, in the range of 50–200 mg, showed linear pharmacokinetics and do not support CYP2D6 inhibition. Furthermore, substrates of the enzyme (MDMA and MDA) could be responsible for pharmacokinetic interactions, leading to acute toxic and neurotoxic effects.

Furthermore, it has been described that the disposition of MDMA is mainly affected by the CYP2D6 polymorphism. However, this influence may be less relevant due to the effect of the self-inhibition mechanism of MDMA on this enzyme and the contribution of other isoenzymes of cytochrome P450 in the metabolism process [22,23,24]. When assessing this effect on pharmacokinetics, it was observed that poor metabolizers achieved higher maximum concentrations of MDMA (+15%) and MDA (+50%), but lower concentrations of HMMA (−50–70%) compared to extensive metabolizers. These differences may have implications in pharmacological effects and toxicity, since poor metabolizers experience higher and faster increases in blood pressure and subjective effects [23]. In addition, higher concentrations of HHMA could lead to the increased formation of some quinones, which are involved in the mechanisms of serotoninergic neurotoxicity [25]. Similarly, a potential influence of enzyme polymorphisms on the toxicity of the reactive intermediate methylone metabolite HHMC can be hypothesized. However, it is still unknown if these effects also occur after methylone administration and further studies to verify this hypothesis are needed.

Considering the few methylone deaths reported in the literature and the fact that methylone exhibits linear pharmacokinetics, it is possible that dose-response proportionality protects against overdose. These results must be confirmed in large samples of subjects and doses in future studies.

## 4. Materials and Methods

### 4.1. Chemicals and Materials

MDMA, MDA, HMMA, HMA, methylone and HMMC standards were purchased from Cerilliant (Round Rock, TX, USA). Deuterated internal standards (ISTD) MDMA-d5, MDA-d5 and methylone-d3 were acquired from Cayman Chemical (Ann Arbor, MI, USA). Standards were stored at −20 °C until analysis. LC-MS grade water, methanol, acetonitrile, formic acid, chloroform and ethyl acetate were obtained from Carlo Erba (Cornaredo, Italia). Ammonium hydroxide (25% purity) and hydrochloric acid (37% purity) were purchased from Honeywell Fluka™ (Morristown, NJ, USA).

### 4.2. Calibrators and Quality Control (QC) Solutions

Two different aliquots (I and II) from each standard stock solution were prepared. Calibrators were made from aliquot I standard stock solution that contained all analytes at 10 and 100 ng/mL in acetonitrile. Aliquot II standard stock solutions (10 and 100 ng/mL) were used to prepare quality controls (QCs). ISTD stock solution was prepared with the same procedure mentioned above at 100 and 1000 ng/mL concentrations and stored in glass vials at −20 °C until use. Blank human plasma was provided by the University storehouse. Plasma was evaluated by in-house routine chromatography–mass spectrometry methods to ensure the absence of methylone and MDMA and their metabolites and the most prevalent drugs of abuse and pooled for the preparation of calibrators and QC.

Based on an initial semi-quantitative analysis of authentic plasma samples, the following calibrators were prepared: 2.5, 5, 25, 50, 250 and 500 ng/mL for methylone and MDMA, and 0.5, 1, 10, 25, 50 and 100 ng/mL for HMMC, MDA, HMA and HMMA. MDMA and methylone low-, medium-, and high-QC samples were 7.5, 150 and 350 ng/mL, respectively, and HMMC, MDA HMA and HMMA low-, medium-, and high-QC samples were 1.5, 20 and 80 ng/mL, respectively.

### 4.3. Controlled Methylone and MDMA Administration to Humans

A randomized, cross-over, placebo-controlled, double-blind study was conducted using 12 male volunteers (mean age: 23 years old (range 22–24); mean weight: 70,2 (range 60.4–87 kg)) at the Hospital Universitari German Trias i Pujol, Institut d’Investigatiò en Ciènces de la Salut Germans Trias i Pujol, in Badalona, Spain. All participants have recreational experience with psychostimulants, such as cocaine, amphetamines, MDMA and synthetic cathinones. Each subject participated in three experimental sessions, during which they received single oral doses of 50, 100, 150 or 200 mg of methylone, 100 mg of MDMA or a placebo (dextromaltose) in each session. For safety, higher doses were administered after lower doses showed good tolerability. Blood samples were collected in lithium heparin vacutainer tubes before and 0.25, 0.5, 0.75, 1, 1.5, 2, 3, 4, 6, 8, 10 and 24 h after dosing. Blood was immediately centrifuged at 3500 rpm for 10 min and the obtained plasma was stored at −20 °C until analysis (around 2–4 months after collection).

The 12 subjects were divided into 4 study groups with 3 drug administration sessions, each separated by a washout period of 5 to 7 days. MDMA capsules contained 100 mg of MDMA and the placebo capsules contained dextromaltose.

Methylone capsules contained 50 mg methylone to administer 50, 100, 150, or 200 mg of methylone. Participants received 5 capsules (containing active substance or placebo) each session so the participants and clinical staff were unaware of the dose. The clinical trial was approved by the local human research ethics committee (CEI-HUGTiP ref. PI-19-082) to investigate the potential for abuse and human pharmacology of methylone. The study was registered on ClinicalTrials.gov (number NCT05488171). It was conducted according to the Declaration of Helsinki recommendations and Spanish rules about clinical investigation. All the participants were informed, both orally and in writing, and signed informed consent prior to inclusion. Prior to study sessions, the participants underwent general medical examination, including blood and urine chemistry tests. Measures of the pharmacological effects were collected (not presented in this manuscript).

### 4.4. Sample Preparation

Briefly, 10 μL of the 100 ng/mL internal standard mixture (methylone-d3, MDA-d5 and MDMA-d5) 2 μL of 2% NH_3_ in H_2_O (pH 9), and 2 mL chloroform: ethyl acetate 9:1 (*v*/*v*) were added to 100 μL of plasma and the tubes were stirred using a roller mixer for 10 min and centrifuged at 3500 rpm for 5 min. Supernatants were placed into clean tubes, 100 μL of acidic methanol (1% HCl) was added to prevent evaporative losses, and the samples were dried under nitrogen for approximately 30 min. Samples were reconstituted in 100 μL of mobile phase A:B (95:5) (0.1% formic acid in water:acetonitrile) and transferred into autosampler vials, prior to injection of 1 μL onto the chromatographic system.

### 4.5. Ultra-High-Performance Liquid Chromatography Tandem Mass Spectrometry (HPLC-MS/MS) Analysis

Analyte separation was achieved on an HPLC 1290 Infinity II (Agilent Technologies Italia S.p.a., Milan, Italy) coupled to a mass spectrometer (6470A Triple Quadrupole LC-MS), equipped with an electrospray ionization source (ESI) operating in the positive mode. Data were acquired with MassHunter^®^ Workstation Quantitative Analysis 10.0 Software (Agilent). The optimization process was conducted automatically with the “MassHunter Optimizer” tool provided by Agilent and manually confirmed. Specifically, the “MassHunter Optimizer” automates the selection of the best precursor ions, the optimization of the fragmentor voltage for each precursor ion, the selection of the best product ions, and optimization of collision energy values for each compound transition. Data were acquired with MassHunter^®^ Workstation Quantitative Analysis 10.0 Software (Agilent).

Separation was performed on a Kinetics^®^ 2.6 µm Phenyl-Hexyl column from Phenomenex^®^ (100 mm × 2.1 mm). Run time was 6 min with a gradient mobile phase composed of 0.1% formic acid in water (A) and acetonitrile (B) at a flow rate of 0.4 mL/min. The initial conditions were 5% B, held for 1 min, increased gradually to 50% B within 2 min, increased to 95% B within 4.0 min, decreased to 5% B and held for 6 min. The autosampler and column oven temperatures were 10 °C and 37 °C, respectively.

The mass spectrometer operated in the scheduled multiple reaction monitoring (MRM) mode, with two transitions for each analyte and ISTD (Table 3).

MS parameter settings were optimized by infusing neat standards (100 ng/mL) individually in methanol and increasing the cone voltage and collision energy. Scan speed (dwell time) was 0.023 s. ESI conditions were optimized as follows: the capillary voltage was 3500 V, source temperature was 300 °C, cone gas flow rate was 10 L/min and desolvation gas flow rate was 12 L/min.

### 4.6. Method Validation

Analytical bias, imprecision, limit of detection (LOD), lower limit of quantification (LLOQ), linearity, carryover, matrix effect (ME), recovery (RE), and dilution integrity were assessed during the method validation following recommendations from the Organization of Scientific Area Committees (OSAC) for Forensic Science, USA [13]. Preliminary experiments with eight calibrator concentrations revealed the most appropriate calibration model.

#### 4.6.1. Linearity

Five calibration curves were established on five separate days with six calibrators from the lower (LLOQ) to the upper limit of quantification (ULOQ), which were calculated by linear least squares regression for each analyte and by performing a Mandel test [26]. Calibrators were required to quantify within ±15% of the target concentration (±20% for LLOQ) and the coefficient of determination had to be higher than or equal to 0.99. Quantifying/confirming transition ratios were required to be within ±20% of the average calibrator transition ion ratio.

#### 4.6.2. Limit of Detection and Quantification

LOD was tested by fortifying 5 different sources of blank plasma at the LLOQ and diluting the sources 2-, 5-, 10-, and 20-fold with blank plasma. For each analyte, the LOD was defined as the lowest concentration at which a peak eluted within ±0.1 min of the average calibrator retention time, with a signal/noise ratio higher than or equal to 3 for both transitions and a quantifying/confirming transition ratio within ±20% of the average calibrator ratio.

LLOQ was tested by fortifying 5 different sources of blank plasma. For each analyte, the LLOQ retention time had to be within ±0.1 min of the average calibrator retention time and quantify within ±20% of the target concentration. In addition, quantifying/confirming transition ratios were required to be within ±20% of the average calibrator ratio.

#### 4.6.3. Carryover

Lack of carryover was verified in triplicate by injecting a blank plasma sample fortified with the analytes at 5 times the ULOQ, followed by a negative sample. Carryover was negligible if no peak eluted within ±0.1 min of the average calibrator retention time, with a signal/noise ratio higher than 3 in the negative samples.

#### 4.6.4. Interferences

Matrix interferences were evaluated with 5 different blank plasma samples. Interferences were negligible if no peak eluted within ±0.1 min of the average calibrator retention time with a signal/noise ratio higher than 3. Moreover, blank plasma samples were analyzed for possible exogenous drug interferences. For this, fortified matrix samples were spiked with other sympathomimetic amines (ephedrine and pseudoephedrine), and cathinones (4-dimethylmethcathinone hydrochloride (3,4-DMMC HCl), 4-methyl-alpha-pyrrolidinoexanophenone (3,4-MDPHP), 3-chloromethcathinone hydrochloride (3-CMC HCl), 3-fluoro-alpha-pyrrolidinopentiophenone (3-fluoro-alpha-PVP), 3-methylmethcathinone hydrochloride (3-MMC HCl), 4-bromomethcathinone hydrochloride (4-BMC HCl), 4-ethylethcathinone hydrochloride (4-EEC HCl), 4′-fluoro-alpha-pyrrolidinohexanophenone hydrochloride (3-fluoro-alpha-PVP HCl), 4-fluoromethcathinone metabolite hydrochloride (4-FMC metabolite HCl), 4-hydroxy-3-methoxy methcathinone (HMMC), 4-methylethcathinone metabolite hydrochloride (4-MEC metabolite HCl), benzedrone hydrochloride (4-MBC HCl), 3,4-methylenedioxy-N-ethylcathinone hydrochloride (ethylone HCl) and β-keto-1,3-benzodioxolyl-N-ethylbutanamine hydrochloride (futhylone HCl)) at 3 different concentrations (10, 50 and 100 ng/mL). A signal/noise ratio no greater than 3 at ±0.1 min of the analytes’ retention period (which ranged from 0.73 to 2.31 min) for the quantitative and qualitative ions was the requirement for acceptability.

#### 4.6.5. Dilution Integrity

Dilution integrity was assessed in five replicates by fortifying blank plasma with analytes at 2 times the ULOQ. Samples were diluted two-, five-, ten- and twenty-fold in blank plasma before analysis and were required to quantify within ±20% of the target concentration with a quantifying/confirming transition ratio within ±20% of the average calibrator ratio.

#### 4.6.6. Stability

Analyte stability was assessed in plasma at room temperature and at +4 °C for 24 h, in plasma following 3 freeze/thaw cycles (−20 °C), and in the LC reconstitution solvent 24 h after extraction and storage in the autosampler (+10 °C). Internal standards were added immediately before extraction. Stability was assessed in 4 replicates at QC1, QC2, and QC3 concentrations.

Room temperature samples and refrigerated samples were analyzed after 24 h. Processed sample stability was measured by extracting low- and high-QC samples (n = 3), combining reconstituted samples, dividing them into different autosampler vials, and immediately analyzing them using the instrument. Ratios of the peak area of the analyte to the internal standard were calculated and triplicates were averaged for each concentration to establish the time zero response. Vials with extracted samples remained on the autosampler (4 °C) and were re-injected at 24 h and the calculated ratios of the peak areas of the analyte to the internal standard were compared to time zero. Finally, mid-term stability was assessed by re-analyzing 3 replicates at QC1, QC2, and QC3 concentrations 1, 2 and 4 months after QC sample preparation and storage at −20 °C.

Analytes were considered stable if the observed concentrations were within ±20% of the target concentration with a quantifying/confirming transition ratio within ±20% of the average calibrator ratio of the target.

#### 4.6.7. Matrix Effect and Recovery

Matrix effect and recovery were evaluated with blank plasma from 5 different sources fortified with the analytes at QC1, QC2, and QC3 concentrations. Three sample sets were prepared, with set A blank plasma fortified with the analytes before extraction, set B blank plasma extracted and fortified with the analytes immediately before evaporation, and set C neat standards in the LC reconstitution solvent. For each analyte, ion suppression or enhancement was calculated by dividing the mean LC-MS/MS peak area of set A by the mean analyte peak area of set B. Ion suppression or enhancement was calculated by dividing the mean LC-MS/MS peak area of set B by the mean analyte peak area of set C, minus 1. The samples were required to quantify within ±20% of the target concentration with a quantifying/confirming transition ratio within ±20% of the average calibrator ratio. Accuracy and imprecision were calculated for each matrix type at the QC concentrations. Acceptable criteria were ±30% of the target and 20% CV for accuracy and imprecision, respectively.

#### 4.6.8. Data Analysis and Statistics

Data from pharmacokinetics experiments were analyzed and graphically displayed using GraphPad Prism 9 (version 9.4.0 (653), San Diego, CA, USA). Plasma pharmacokinetics data were further analyzed using WinNonlin to determine the non-compartmental pharmacokinetics constants.

## 5. Conclusions

We developed and validated the first analytical method for simultaneously quantifying methylone, MDMA and their principal metabolites (one metabolite for methylone and three for MDMA) in human plasma samples, starting with controlled administration at different doses. The extraction and analysis method was simple, fast and effective for the detection of all analytes of interest and was fully validated. Methylone plasma concentrations increased in a linear dose-related manner, in contrast to MDMA. The elimination half-life of methylone was 6–7 h.

Considering the results obtained, further studies are needed to better understand the pharmacokinetics of synthetic cathinones and to analyze all the metabolites from different compounds, including methylone metabolites, and the impact that these new drugs might have on human health. The analytical method developed in this study will certainly be useful for the analysis of future methylone cases.

## Figures and Tables

**Table 1:** Method validation parameters and Mandel’s fitting test (*p*-value and Fcrit95%) for analytes under investigation in plasma sample.

	Linear Range ng/mL	r²	*p*-Value	F_crit95%_	LOD ng/mL	LOQ ng/mL	QC ng/mL			Accuracy ng/mL			Intra-Day Imprecision CV (%)			Inter-Day Imprecision CV (%)			Recovery (%)			Matrix Effect (%)		
							L	M	H	L	M	H	L	M	H	L	M	H	L	M	H	L	M	H
Methylone	2.5–500	0.9973	0.126	2.495	0.5	2.5	7.5	150	350	120	95.8	117	1.4	3.5	5.0	3.7	5.9	3.9	112	103	96	7.3	−5.2	1.9
HMMC	0.5–100	0.9892	0.741	0.110	0.1	0.5	1.5	20	80	102	96.5	101	2.5	4.0	6.1	2.1	7.1	2.2	111	105	107	3.1	−6.0	0.6
MDMA	2.5–500	0.9985	0.857	0.032	0.5	2.5	7.5	150	350	93.0	93.5	98.6	1.7	3.9	1.7	2.2	4.6	2.2	113	102	103	1.5	−1.8	1.2
MDA	0.5–100	0.9998	0.665	0.191	0.1	0.5	1.5	20	80	98.2	94.2	91.8	2.3	0.9	6.1	4.4	3.2	0.7	108	104	103	−7.7	−4.6	2.1
HMA	0.5–100	0.9999	0.057	0.900	0.1	0.5	1.5	20	80	99.2	93.9	112	1.9	5.8	0.9	3.0	5.7	2.7	119	102	99.4	−4.0	−2.1	−3.8
HMMA	0.5–100	0.9972	0.221	1.136	0.1	0.5	1.5	20	80	102	107	98.8	1.4	2.2	4.3	4.6	7.0	1.1	114	104	99.7	−8.5	−6.6	−5.2

Abbreviations: HMMC, 4-hydroxy-3methoxymethcathinone; MDMA, 3,4-methylendioxymethamphetamine; MDA, 3,4-methylendioxyamphetamine; HMA, 4-hydroxy-3-methoxyamphetamine; HMMA, 4-hydroxy-3methoxymethamphetamine; LOD, limit of detection; LOQ, limit of quantification; L, low-quality control; M, medium-quality control; H, high-quality control; CV, coefficient of variation.

**Table 2:** Pharmacokinetic parameters for plasma methylone, HMMC, MDMA, MDA, HMMA and HMA.

Analytes	Dose mg (Subjects)	Cmax ng/mL	Tmax h	AUC0–10	AUC0–24	T1/2	Clast ng/mL
				h·ng/mL	h·ng/mL	h	
Methylone	50 (n = 3)	153.0	1.5	729.2	1042.8	5.8	8.0
	100 (n = 6)	304.0	2.5	1596.2	2441.2	6.4	18.8
	150 (n = 5)	355.0	2.0	2245.6	3524.4	6.9	38.6
	200 (n = 7)	604.0	2.0	3329.1	5067.9	6.4	47.8
HMMC	50 (n = 3)	15.3	1.0	36.6	47.7	5.8	0.5
	100 (n = 6)	24.3	0.9	65.8	87.9	4.5	0.4
	150 (n = 5)	25.9	1.0	75.5	109.4	7.2	1.3
	200 (n = 7)	42.1	1.5	136.3	174.7	6.3	1.0
MDMA	100 (n = 3)	66.1	2.0	468.2	888.6	12.3	16.6
MDA	100 (n = 3)	21.9	4.0	135.9	290.8	44.7	9.6
HMMA	100 (n = 3)	85.6	3.0	240.8	294.1	5.4	1.8
HMA	100 (n = 3)	20.8	3.0	62.3	71.0	5.3	0.7

Abbreviations: HMMC, 4-hydroxy-3methoxymethcathinone; MDMA, 3,4-methylendioxymethamphetamine; MDA, 3,4-methylendioxyamphetamine; HMA, 4-hydroxy-3-methoxyamphetamine; HMMA, 4-hydroxy-3methoxymethamphetamine; Cmax, maximum concentration; Tmax: time to reach maximum concentration; AUC, area under the curve; T1/2, elimination half-life; Clast, last concentration at 24 h.

**Table 3:** Mass spectrometry parameters for analytes and internal standards with positive ionization.

Analytes	Molecular Mass	Precursor Ion	Product Ion *m/z*	Retention Time Min	CE
	g/mol	*m/z*			eV
MDMA	193.2	194.2	105.0	2.16	25
			**163.0**	2.16	9
MDMA-d_5_	198.2	199.2	107.1	2.15	29
			**165.1**	2.15	13
MDA	179.2	180.2	77.1	1.91	45
			**105.1**	1.91	25
MDA-d_5_	198.2	199.2	110.1	1.90	24
			**168.1**	1.90	8
HMMA	231.7	232.0	105.1	1.07	25
			**165**	1.07	8
HMA	181.0	182.0	105.1	0.87	24
			**165.1**	0.87	8
Methylone	207.2	208.2	132.1	1.79	29
			**160.1**	1.79	17
Methylone-d_3_	210.2	211.2	135.1	1.78	16
			**163.1**	1.78	16
HMMC	209.2	210.2	192.1	1.01	12
			**160.1**	1.01	16

Abbreviations: HMMC, 4-hydroxy-3methoxymethcathinone; MDMA, 3,4-methylendioxymethamphetamine; MDA, 3,4-methylendioxyamphetamine; HMA, 4-hydroxy-3-methoxyamphetamine; HMMA, 4-hydroxy-3methoxymethamphetamine; CE, collision energy. Quantification ions are in bold.

## Data Availability

Data are contained within the article.
